# Monocyte, macrophage and mast cell-derived PDGF control inflammation, tissue remodelling and autoimmunity in the pathophysiology of Graves’ ophthalmpathy

**DOI:** 10.1186/1479-5876-9-S2-P8

**Published:** 2011-11-23

**Authors:** Leendert van Steensel, Dion Paridaens, P Martin van Hagen, Robert WAM Kuijpers, Willem A van den Bosch, Hemmo A Drexhage, Herbert Hooijkaas, Willem A Dik

**Affiliations:** 1 Dept. of Immunology, Erasmus MC, University Medical Center, Rotterdam, The Netherlands; 2 Dept. of Opthalmology, Erasmus MC, University Medical Center, Rotterdam, The Netherlands; 3 Rotterdam Eye Hospital, Rotterdam, The Netherlands

## Introduction

Orbital fibroblast activation leading to excessive proliferation, hyaluronan production and cytokine production is critical in the development of Graves’ ophthalmopathy (GO). Various cell types produce factors such as cytokines and growth factors that orchestrate orbital fibroblast (OF) activity. Furthermore, TSHR stimulating antibodies also stimulate OF activity. However, more insight into the interrelationships between the different cell types, cytokines, growth factors, TSHR autoantibodies and orbital TSHR expression is needed in GO.

## Aim

To examine the relationship between platelet-derived growth factor (PDGF), OF activation and TSHR expression and autoantibodies in GO.

## Methods

Expression of PDGF family members was determined by RQ-PCR, Western Blot and immunohistochemistry in GO and control orbital tissue. The effect of PDGF-AA, PDGF-AB and PDGF-BB was determined on OF proliferation, IL-6 production and hyaluronan production. Moreover, the effect of PDGF on TSHR expression by OF and their subsequent susceptibility for Graves’ Disease immunoglobulins (GD-IgG) was determined.

## Results

PDGF-A and PDGF-B chains were increased in GO orbital tissue. Immunohistochemistry revealed that this was due to production by monocytes, macrophages and mast cells. PDGF induced proliferation, cytokine production and hyaluronan production by OF. Furthermore, PDGF increased TSHR expression on OF and enhanced their susceptibility to TSH and GD-IgG with regard to IL-6 and hyaluronan production. These effects of TSH and GD-IgG were inhibited by a cAMP inhibitor and by a TSHR blocking antibody, suggesting involvement of TSHR signaling. Remarkably, PDGF-BB has the most pronounced effect on OF, while PDGF-AA showed the least effect.

## Conclusion

Monocytes, macrophages and mast cells secrete PDGF, of which especially PDGF-BB and PDGF-AB stimulate OF activity, thereby enhancing orbital inflammation, tissue remodeling and TSHR autoantibody-driven autoimmunity in GO patients.

